# Asperpyrone-Type Bis-Naphtho-γ-Pyrones with COX-2–Inhibitory Activities from Marine-Derived Fungus *Aspergillus niger*

**DOI:** 10.3390/molecules21070941

**Published:** 2016-07-20

**Authors:** Wei Fang, Xiuping Lin, Jianjiao Wang, Yonghong Liu, Huaming Tao, Xuefeng Zhou

**Affiliations:** 1 Hubei Biopesticide Engineering Research Center, Hubei Academy of Agricultural Science, Wuhan 430064, China; fangw111@163.com; 2 Key Laboratory of Tropical Marine Bio-Resources and Ecology, Guangdong Key Laboratory of Marine Materia Medica, South China Sea Institute of Oceanology, Chinese Academy of Sciences, Guangzhou 510301, China; xiupinglin@hotmail.com (X.L.); yonghongliu@scsio.ac.cn (Y.L.); 3 Department of Pharmacy, Medical College, Wuhan University of Science and Technology, Wuhan 430065, China; jianjiao1102@163.com; 4 School of Traditional Chinese Medicine, Southern Medical University, Guangzhou 510515, China

**Keywords:** bis-naphtho-γ-pyrones, *Aspergillus niger*, COX-2–inhibitory, HPLC/MS

## Abstract

Bis-naphtho-γ-pyrones (BNPs) are an important group of aromatic polyketides derived from fungi, and asperpyrone-type BNPs are produced primarily by *Aspergillus* species. The fungal strain *Aspergillus niger* SCSIO Jcsw6F30, isolated from a marine alga, *Sargassum* sp., and identified according to its morphological traits and the internal transcribed spacer (ITS) region sequence, was studied for BNPs secondary metabolisms. After HPLC/MS analysis of crude extract of the fermentation broth, 11 asperpyrone-type BNPs were obtained directly and quickly by chromatographic separation in the extract, and those isolated asperpyrone-type BNPs were structurally identified by NMR and MS analyses. All of the BNPs showed weak cytotoxicities against 10 human tumor cells (IC_50_ > 30 μM). However, three of them, aurasperone F (**3**), aurasperone C (**6**) and asperpyrone A (**8**), exhibited obvious COX-2–inhibitory activities, with the IC_50_ values being 11.1, 4.2, and 6.4 μM, respectively. This is the first time the COX-2–inhibitory activities of BNPs have been reported.

## 1. Introduction

Endophytic fungi from various marine algal hosts are increasingly being considered as sources of pharmaceutical bioactive compounds, especially *Aspergillus* and *Penicillium*, as the most commonly isolated fungal endophytes [1]. *Aspergillus niger*, probably the most common of the black aspergilla, exhibits a remarkably productive metabolism, which has made it one of the most important microorganisms used for industrial fermentations [2].

Bis-naphtho-γ-pyrones (BNPs), also called dimeric naphtho-γ-pyrones or bis-naphthopyran-4-ones, are an important group of aromatic polyketides derived from fungi. They have a variety of biological activities including antioxidant, antitumor, antimicrobial, and tyrosine kinase and HIV-1 integrase inhibition properties, demonstrating their potential applications in medicine and agriculture. About 60 BNPs have been reported from filamentous fungi in the past few decades, such as *Aspergillus* sp. and *Fusarium* sp. [3]. BNPs can be divided into three types, named chaetochromin, asperpyrone, and nigerone types, according to the diaryl bond connection. It is interesting that both asperpyrone and nigerone types of BNPs are produced primarily by *Aspergillus* species, where chaetochromin types are not distributed [4].

Cyclooxygenase (COX) enzymes are the pharmacological targets of nonsteroidal anti-inflammatory drugs (NSAIDs). COX-2, as a well-established target, is an inducible enzyme in which expression is activated by cytokines, mitogens, endotoxins and tumor promoters. The anti-inflammatory and analgesic properties of traditional NSAIDs are primarily due to the inhibition of COX-2 [5].

**Figure 1:**
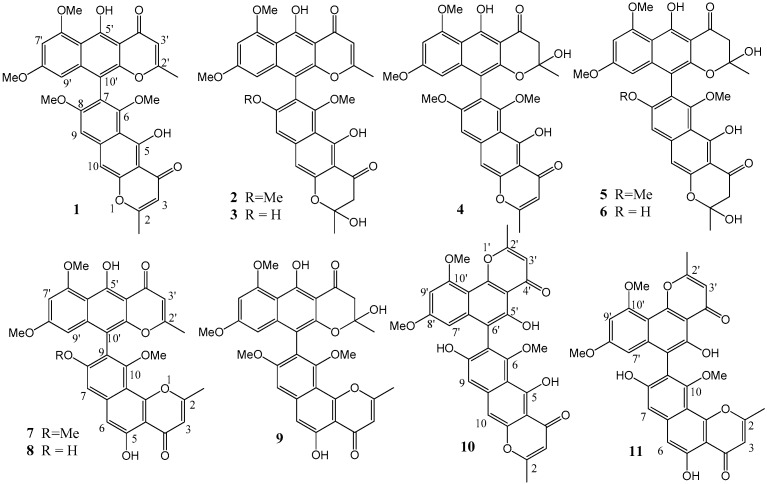
Structures of compounds **1**–**11**.

During our continuous studies of screening for COX-2 inhibitors in marine-derived fungi, BNPs, as the main secondary metabolites of a marine alga-derived *Aspergillus* fungus, showed obvious COX-2–inhibitory activities. In the present paper, 11 BNPs (**1**–**11**) (Figure 1) were obtained quickly by chromatographic separation, in the fermentation broth of the strain *Aspergillus niger* SCSIO Jcsw6F30, guided by HPLC/MS analysis. Those asperpyrone-type BNPs were structurally identified by NMR and MS analyses and tested for their cytotoxic activities against several human tumor cells and for their COX-2–inhibitory activities.

## 2. Results

### 2.1. Characterization and Identification of Isolated Strain SCSIO Jcsw6F30

**Figure 2:**
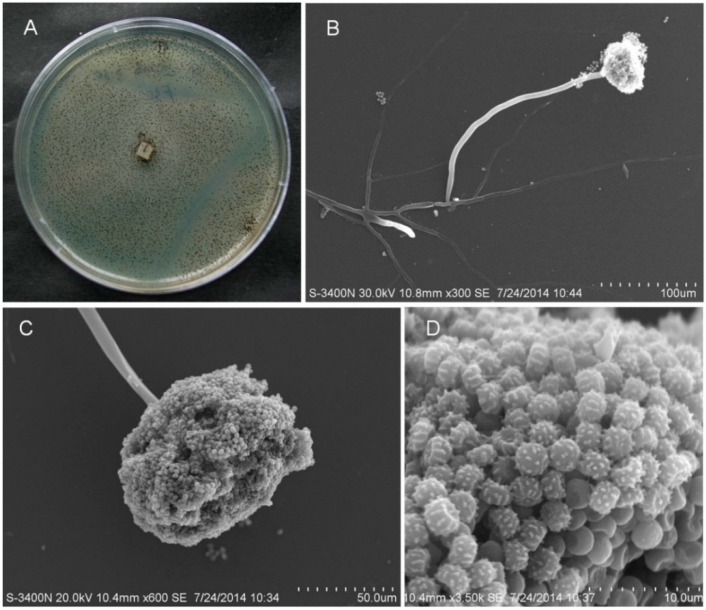
Colony appearance and micromorphology of *A. niger* SCSIO Jcsw6F30. (**A**) Colony appearance after seven days at 25 °C (MB); (**B**,**C**) Conidiophores after seven days at 25 °C under SEM; (**D**) Conidia as seen using SEM.

Fungal strain SCSIO Jcsw6F30 was isolated and purified from a marine alga, *Scagassum* sp., After 7 days of growth on Medium B agar medium at 25 °C; colonies were about 90 mm in diameter, and showed good sporulation. The spores were black, whereas the mycelia were white (Figure 2A).

Scanning electron microscopy (SEM) revealed that the mycelia were composed of branched, septate, smooth-walled hyphae 2.6 μm to 3.7 μm wide (mean = 3.4 μm). Conidial heads were radiated and subglobose. Stipes were 243 μm to 485 μm long (mean = 364 μm), were 5.9 μm to 9.2 μm wide (mean = 7.1 μm), and had smooth walls. Conidia were black and wheel-shaped when mature, measuring 1.5–2.4 (mean = 1.9) × 2.8–3.5 (mean = 3.1) µm, and smooth or roughened (Figure 2B–D). A teleomorphic state was not observed.

**Figure 3:**
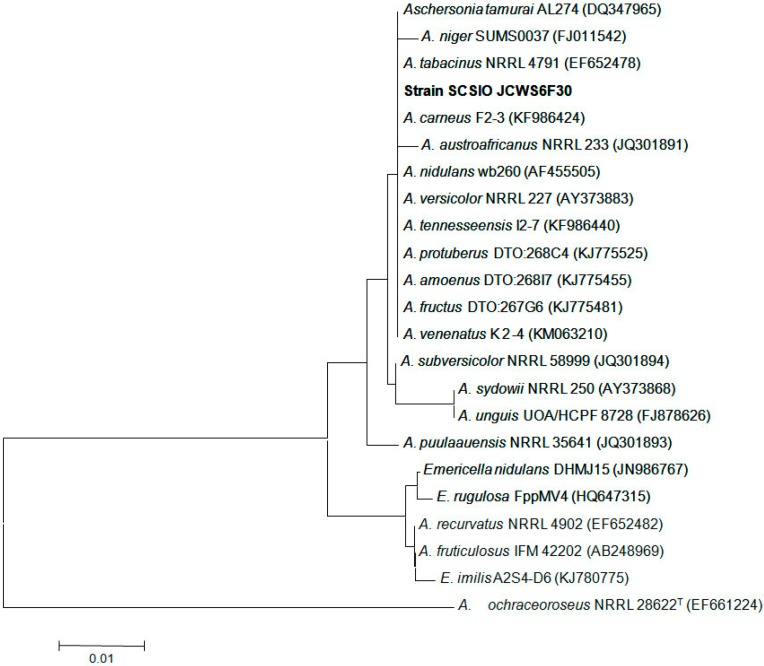
The neighbor-joining tree based on ITS1-5.8S-ITS2 sequences, showing the phylogenetic relationship between strain SCSIO Jcsw6F30 and related species. GenBank accession numbers are given in parentheses. Bar: 1% sequence divergence.

The ITS1-5.8S-ITS2 sequence region (521 basepairs, accession number KT119567) of strain SCSIO Jcsw6F30 was amplified by PCR and sequenced. A phylogenetic tree was constructed, using the neighbor-joining method based on the similarity of a 461 bp consensus length of the ITS1-5.8S-ITS2 sequence (Figure 3). Strain SCSIO Jcsw6F30 was found to belong to a clade related to *A. cameus* F2-3, *A. tabacinus* NRRL 4791 and *A. niger* SUMS0037 et al. in the tree, with sequence identities of 99.8%, 99.6% and 99.2%, respectively. The properties of the culture and the morphology of SCSIO Jcsw6F30 were consistent with those of *A. niger* as previously reported [6]. The ITS phylogenetic analyses confirmed that the fungus strain SCSIO Jcsw6F30 belonged to *A. niger*, and was designated as *A. niger*.

### 2.2. HPLC/MS Analysis of Crude Extract of the Fermentation Broth

Naphtho-γ-pyrones present very characteristic UV/vis spectra, two strong absorbance peaks at 215–230 nm, 270–285 nm, and two weak absorbance peaks at 330–340, 395–410 nm, because of their fully conjugated system. The absorption spectra mainly depend on the isomeric form and the polymerization degree [2]. Moreover, monomeric or dimeric naphtho-γ-pyrones could be determined by MS data.

**Figure 4:**
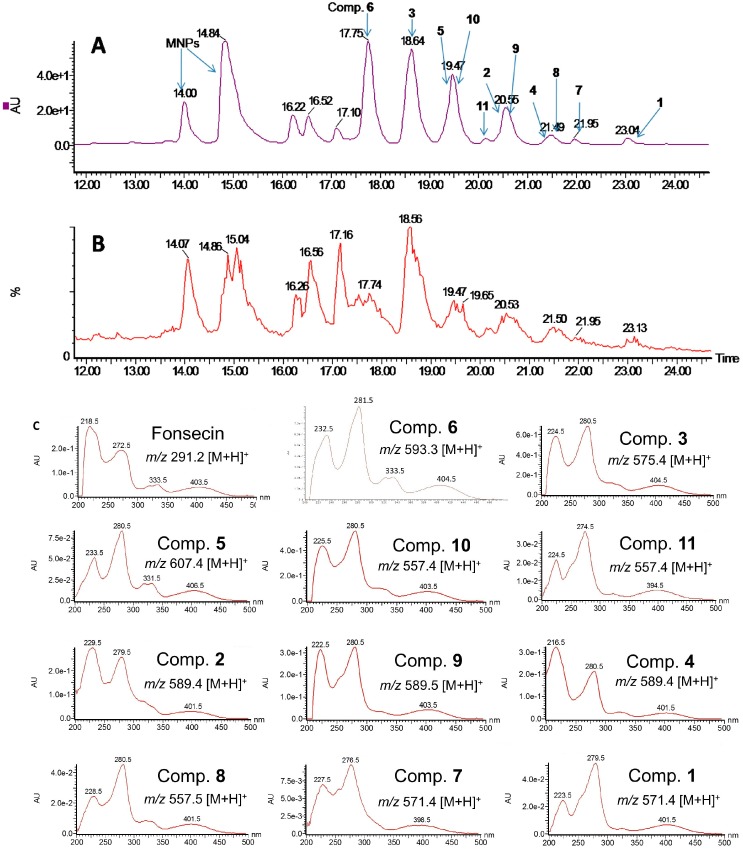
HPLC/MS data of the extract of the fermentation broth. (**A**) Main part of the HPLC (diode array) chromatogram of the extract; (**B**) Main part of the total ion chromatogram (TIC) of the extract; (**C**) All of the 11 BNPs (**1**–**11**) had similar UV absorption spectra (UV spectra are placed in order of retention time in chromatogram A), together with their molecular ion given by MS.

The HPLC/MS data showed at least 11 BNPs signals (R_t_ 17.5–23.5 min), according to the characteristic UV/vis spectra and molecular ion peaks (*m/z* 557–607), although their exact structures remained unknown (Figure 4). For example, the UV spectrum [232.5 (71%), 281.5 (100%), 333.5 (23%), 404.5 (17%)] and molecular ion peaks (*m/z* 593.3 [M + H]^+^, 615.5 [M + Na]^+^) of the HPLC peak at R_t_ 17.75 min suggest it is a BNP compound with the molecular formula C_31_H_28_O_12_. The HPLC peak at R_t_ 14.00 min showed a similar UV spectrum; however, the molecular ion peaks (*m/z* 291.2 [M + H]^+^) suggest they are monomeric naphtho-γ-pyrones (MNPs) (Figure 4). The molecular formulas of 11 BNPs were suggested to be C_32_H_26_O_10_ (**1**), C_32_H_28_O_11_ (**2**), C_31_H_26_O_11_ (**3**), C_32_H_28_O_11_ (**4**), C_32_H_30_O_12_ (**5**), C_31_H_28_O_12_ (**6**), C_32_H_26_O_10_ (**7**), C_31_H_24_O_10_ (**8**), C_32_H_28_O_11_ (**9**), C_31_H_24_O_10_ (**10**), and C_31_H_24_O_10_ (**11**), respectively, according to the molecular weight and the molecular rules of BNPs.

### 2.3. Structural Elucidation

The ^1^H-NMR spectra of the isolated BNPs were obtained on a Bruker AVANCE-500 spectrometer. The ^1^H-NMR characteristics of those BNPs showed they were asperpyrone-type BNPs with different linkages. The diagnostic signals at δ_H_ 14.86 (s) and 15.27 (s) for the two phenolic OH groups (5, 5’-OH) in its ^1^H-NMR spectrum suggested that compound **1** was linked by two linear monomeric aromatic polyketide moieties via C-7/C-10’, like the linkages of compounds **2**–**6**. Compound **7** was suggested to have C-9/C-10’ linkages with angular and linear monomeric moieties, according to the two phenolic OH signals at δ_H_ 12.86 (s) and 15.27 (s), as well as compounds **8**–**9** [7]. Compounds **10** and **11** were considered as C-7/C-6’ and C-9/C-6’ linkages because of their different chemical shift of two phenolic OH groups (δ_H_ 14.80 and 13.44 of **10**, δ_H_ 12.74 and 13.47 of **11**) [8]. The AB system of the methylene protons (such as δ_H_ 3.03 and 3.07, *J* = 17.0 Hz, in **2**) in some ^1^H-NMR spectra suggested that the C2/C3 or C2’/C3’ double bonds might be hydrogenated in those compounds (compounds **2**–**6** and **9**) [9].

Compounds **1**–**11** were identified as aurasperone A (**1**) [7], fonsecinone D (**2**) [10], aurasperone F (**3**) [11], fonsecinone B (**4**) [12], aurasperone B (**5**) [10], aurasperone C (**6**) [11], fonsecinone A (**7**) [7], asperpyrone A (**8**) [13], fonsecinone C (**9**) [12], asperpyrone D (**10**) [8], asperpyrone E (**11**) [14] by molecular formula given by LC/MS and comparison with their ^1^H NMR (Supplementary Materials Table S1) with those reported.

### 2.4. Bioactivities

These 11 BNPs displayed very weak cytotoxicities (IC_50_ > 30 μM) against 10 human cancer cell lines (K562, A549, Du145, H1975, MCF-7, Huh-7, HL7702, HL60, HeLa, and Molt-4). Aurasperone F (**3**) was showed to be the relative strongest cytotoxic BNP compound among them, with the best inhibitory rates of 38.8%, 41.0%, 44.9%, 46.6%, and 49.3% against HeLa, MCF-7, Molt-4, Huh-7, and H1975, respectively, at the concentration of 30 μM. However, three of these BNPs, aurasperone F (**3**), aurasperone C (**6**), and asperpyrone A (**8**), with a C-8 phenolic OH group in the structure, exhibited obvious COX-2–inhibitory activities, with the IC_50_ values being 11.1, 4.2, and 6.4 μM, respectively (Supplementary Materials Table S2).

## 3. Discussion

BNPs, known as mycotoxins, have been reported with a variety of biological activities including antioxidant, antitumor, antimicrobial, and tyrosine kinase and HIV-1 integrase inhibition properties. Although the activities of three BNPs are much weaker than that of nonsteroidal anti-inflammatory drugs (NSAIDs) Celecoxib, a highly selective COX-2 inhibitor, this is the first time the COX-2–inhibitory activities of BNPs have been reported, presenting a new type of natural product as potential COX-2 inhibitors. Three BNPs with obvious COX-2–inhibitory activities are characterized as C-8 free hydroxyl group and C-10’ (linear naphtho-γ-pyrone) linkages. However, the complete structure-activity relationship is yet to be determined in a future study. The weak cytotoxicities reported in this paper also contribute to these BNPs being potential anti-inflammatory agents. BNP compounds should be screened comprehensively or structurally modified to be optimized as COX-2 inhibitors or anti-inflammatory agents.

BNPs could be produced in large amounts by industrial fungi, such as *Aspergillus niger* [2]. *Aspergillus niger* SCSIO Jcsw6F30, obtained from marine alga *Sargassum* sp., could be considered as a good industrial fungus to produce asperpyrone-type BNPs, according to our research. The LC/MS analysis, together with the quick isolation and structural identification of BNPs by ^1^H-NMR described in this paper, also shows benefits of the industrial application of asperpyrone-type BNPs.

## 4. Materials and Methods

### 4.1. Cultural and Morphological Properties of Strain SCSIO Jcsw6F30

The fungal strain SCSIO Jcsw6F30 was isolated from a marine alga *Scagassum* sp. collected near the Yongxing Island, South China Sea (in July 2012), and grown on Medium B agar at 25 °C [15]. This strain was stored on Medium B agar slants at 4 °C and then deposited at the Key Laboratory of Tropical Marine Bio-Resources and Ecology, CAS (Guangzhou, China).

The cultural properties of strain SCSIO Jcsw6F30 were described after culturing on Medium B agar medium at 25 °C for 7 days. The morphological features of the spores and mycelia in the 7 d cultures grown on Medium B medium (consisting of 15 g malt extract, 10 g sea salt, 15 g agar, 1000 mL distilled water, pH 7.4–7.8) were examined. The samples were observed with a Hitachi S-3400N scanning electron microscope using a previously described cover technique [16,17].

### 4.2. ITS Region Sequence and Phylogenetic Analysis

The mycelia of strain SCSIO Jcsw6F30 cultured in Sabouraud’s Dextrose Broth (consisting of 40 g dextrose, 10 g peptone, 2.5 g NaCl, and 1000 mL distilled water, pH 5.6) were sampled and powdered in a mixer mill after liquid nitrogen was added. DNA was isolated through the Hpure Fungal DNA Kit (Genebase Bioscience Co., Guangzhou, China) according to the manufacturer’s protocol. The ITS region of strain SCSIO Jcsw6F30 was amplified by polymerase chain reaction with the primer pair ITS1–ITS4. The amplified product was purified with a TIANgel mini purification kit (TianGen Biotech, Beijing, China). Pure PCR product was submitted for sequencing together with the primer ITS1 to a commercial service (Shanghai Majorbio Bio-pharm Technology Co., Ltd., Shanghai, China). The derived ITS region sequence was compared against the GenBank database (NCBI) through BLAST-Algorithmus. Similarity analysis was performed using ClustalW program. The phylogenetic tree of strain SCSIO Jcsw6F30 was constructed using neighbor-joining method, as previously described [18]. *Aspergillus ochraceoroseus* NRRL 28622^T^ was used as an outgroup. The nucleotide sequence of the ITS region reported in this article was assigned the GenBank accession number KT119567.

### 4.3. Fermentation and Extraction

The strain was cultured on Medium B agar plates and then incubated for seven days previously described [19]. Seed medium (consisting of 6.25 g maltose, 6.25 g malt extract, 1 g yeast extract, 6.25 g peptone, 1.25 g potassium dihydrogen phosphate, and 1000 mL distilled water, pH 7.0) in 500 mL Erlenmeyer flasks was inoculated with strain Jcsw6F30 and then incubated at 25 °C for 3 days on a rotating shaker (120 rpm). Production medium (the same as the seed medium) in 500 mL flasks was inoculated with 10% seed solution. The flasks were incubated at 28 °C statically. After 7 days, broth from 100 flasks (15 L) was harvested to isolate substances. The broth (15 L) was extracted with 10 L ethyl acetate stirring three times for 30 min. 

### 4.4. HPLC/MS Analysis and Isolation of the Compounds

First 10 Mg crude extract was prepared in 1 mL acetonitrile for HPLC/MS analysis. Two microliters of the extract were subjected to HPLC (a WATERS e 2695 separation module with a WATERS 2996 photodiode array detector). A reverse phase C18 column (2.1 × 150 mm, 3.5 μm, sunfire™, Waters) was employed. The analysis condition was set to a flow rate of 0.3 mL·min^−1^, with a water:acetonitrile step gradient used in the separation method and consisting of 5% acetonitrile for 2 min, gradient to 100% acetonitrile for 25 min, 100% acetonitrile for 5 min, gradient to 5% for 5 min and 5% acetonitrile for 3 min.

MS data were obtained with Waters Quattro ZQ mass spectrometer coupled to the HPLC system. MS parameters: ESI, alternative ion polarity mode, capillary voltage, 3.5 kv, Cone voltage, 45 kv, Desolvation temperature, 300 °C, Desolvation gas flow, 500 L/h, mode, full scan: *m/z* range, 100–1500.

The crude extract was subjected to a silica gel CC and was separated by a linear gradient of petroleum ether (60–90 °C):EtOAc (50:0, 50:1, 20:1, 10:1, 5:1, 2:1, 1:1, and 0:1) to yield eight fractions (fr. I–fr. VIII). HPLC/MS analyses showed those BNPs were distributed in two fractions: fr. VI–VII. Fractions VI–VII were dissolved in methanol and chromatographed over semi-preparative HPLC (Sunfire, Prep C_18_ OBD, 10 mm × 250 mm, 5 µm, 7.5 mL/min) with a gradient solvent system from 20% to 60% CH_3_CN over 30 min, respectively. Eleven BNPs were purified and obtained by repeated semi-preparative HPLC, guided by the HPLC/MS information.

### 4.5. Bioassays

A previously described bioassay test was used to evaluate the cytotoxicities of those BNPs against 10 human cancer cell lines (K562, A549, Du145, H1975, MCF-7, Huh-7, HL7702, HL60, HeLa, and Molt-4) [18]. The isolated compounds were tested for COX-2 inhibitory activity using the COX (ovine) inhibitor screening kit according to the manufacturer’s instructions, as previously described [20]. Celecoxib (Sigma, St. Louis, MO, USA) was used as the positive control with an IC_50_ value of 11.3 nM.

## Supplementary Materials

Supplementary materials can be accessed at: http://www.mdpi.com/1420-3049/21/7/941/s1.

## Abbreviations

The following abbreviations are used in this manuscript:

BNPs	bis-naphtho-γ-pyrones
COX	cyclooxygenase
CC	column chromatography
NSAIDs	nonsteroidal anti-inflammatory drugs
SEM	scanning electron microscopy
HPLC/MS	high performance liquid chromatography/mass spectrometer
NMR	nuclear magnetic resonance
