# Supplementary material for: Asperpyrone-Type Bis-Naphtho-γ-Pyrones with COX-2–Inhibitory Activities from Marine-Derived Fungus Aspergillus niger
Source: Molecules. 2016 Jul 20;21(7):941. doi: 10.3390/molecules21070941 (PMC6273789; doi:10.3390/molecules21070941)
Supplement: Supplementary file 1 [file molecules-21-00941-s001.pdf]

Supplementary Materials: Asperpyrone-Type Bis-Naphtho-γ-Pyrones with COX-2–Inhibitory Activities from Marine-Derived Fungus *Aspergillus niger*

Wei Fang, Xiuping Lin, Jianjiao Wang, Yonghong Liu, Huaming Tao and Xuefeng Zhou

Table S1. ^{1}H NMR spectroscopic data for compounds **1–11**.

No.	δ_H (J in Hz)										
	**1**	**2**	**3**	**4**	**5**	**6**	**7**	**8**	**9**	**10**	**11**
3	6.08 (s)	3.03, 3.07 (d, 17.0)	2.97, 3.05 (d, 17.0)	6.07 (s)	2.97, 3.05 (d,17.0)	2.89, 2.96 (d, 17.0)	6.36 (s)	6.27 (s)	6.37 (s)	6.00 (s)	6.37 (s)
6	/	/	/	/	/	/	7.08 (s)	7.03 (s)	7.07 (s)	/	7.00 (s)
7	/	/	/	/	/	/	7.00 (s)	7.19 (s)	7.02 (s)	/	7.16 (s)
9	7.00 (s)	6.72 (s)	6.65 (s)	7.00 (s)	6.70 (s)	6.60 (s)	/	/	/	7.04 (s)	
10	7.18 (s)	6.86 (s)	7.04 (s)	7.15 (s)	6.86 (s)	6.98 (s)	/	/	/	7.13 (s)	
2-CH_3	2.44 (s)	1.83 (s)	1.80 (s)	2.43 (s)	1.81 (s)	1.74 (s)	2.51 (s)	2.49 (s)	2.51 (s)	2.38 (s)	2.50 (s)
5-OH	14.86 (s)	14.17 (s)	14.27 (s)	14.56 (s)	14.16 (s)	14.17 (s)	12.86 (s)	12.75 (s)	12.80 (s)	14.80 (s)	12.74 (s)
6-OCH_3	3.48 (s)	3.44 (s)	3.39 (s)	3.44 (s)	3.40 (s)	3.35 (s)	/	/	/	3.64 (s)	/
8-OCH_3	3.81 (s)	3.78 (s)	/	3.84 (s)	3.81 (s)	/	3.81 (s)	/	3.86 (s)	/	/
10-OCH_3		/	/		/		3.45 (s)	3.43 (s)	3.42 (s)	/	3.65 (s)
3′	6.01 (s)	6.00 (s)	5.90 (s)	2.94, 2.96 (d, 17.0)	2.94, 3.01 (d, 17.0)	2.81, 2.93 (d, 17.0)	6.03 (s)	6.22 (s)	2.95, 3.01 (d, 17.0)	6.31 (s)	6.34 (s)
7′	6.44 (d, 2.1)	6.43 (d, 2.1)	6.25 (d, 2.1)	6.38 (d, 2.1)	6.37 (d, 2.1)	6.37 (d, 2.1)	6.45 (d, 2.1)	6.34 (d, 2.1)	6.40 (d, 2.1)	6.29 (d, 2.1)	6.28 (d, 2.1)
9′	6.23 (d, 2.1)	6.23 (d, 2.1)	6.23 (d, 2.1)	6.14 (d, 2.1)	6.14 (d, 2.1)	6.14 (d, 2.1)	6.21 (d, 2.1)	5.99 (d, 2.1)	6.16 (d, 2.1)	6.46 (d, 2.1)	6.51 (d, 2.1)
2′-CH_3	2.14 (s)	2.15 (s)	2.16 (s)	1.51 (s)	1.50 (s)	1.46 (s)	2.14 (s)	2.19 (s)	1.49 (s)	2.52 (s)	2.58 (s)
5′-OH	15.27 (s)	15.25 (s)	15.09 (s)	14.82 (s)	14.54 (s)	14.35 (s)	15.27 (s)	15.14 (s)	14.56 (s)	13.44 (s)	13.47 (s)
6′-OCH_3	4.05 (s)	4.04 (s)	3.82 (s)	4.02 (s)	4.02 (s)	3.85(s)	4.06 (s)	3.90 (s)	4.04 (s)	/	/
8′-OCH_3	3.64 (s)	3.66 (s)	3.66 (s)	3.66 (s)	3.66 (s)	3.65 (s)	3.64 (s)	3.69 (s)	3.67 (s)	3.62 (s)	3.64 (s)
10′-OCH_3	/	/	/	/	/		/	/	/	4.00 (s)	4.04 (s)

Table S2. COX-2 inhibitory activities (IC_{50}) of compounds **1–11**.

**Compounds**	**1**	**2**	**3**	**4**	**5**	**6**	**7**	**8**	**9**	**10**	**11**	**Celecoxib**
IC_{50} (μM)	-*	-	11.1	-	-	4.2	-	6.4	-	-	-	0.011

* - means no obvious activities, $IC_{50} > 30$ μM.
